# Retraction Note: Circular RNA circ_103820 suppresses lung cancer tumorigenesis by sponging miR-200b-3p to release LATS2 and SOCS6

**DOI:** 10.1038/s41419-025-08272-x

**Published:** 2025-11-26

**Authors:** Yongbin Chi, Wenlong Zheng, Guangyu Bao, Lifeng Wu, Xiaoxue He, Ruyi Gan, Yan Shen, Xudong Yin, Mingming Jin

**Affiliations:** 1 Medical Laboratory, Gongli Hospital of Shanghai Pudong New Area, Shanghai, China; 2 Department of Clinical Laboratory, Shanghai Pudong Hospital, Fudan University Pudong Medical Center, Shanghai, China; 3 Department of Clinical Laboratory, Affiliated Hospital of Yangzhou University, Yangzhou, China; 4 Department of Laboratory Medicine, The 3rd Affiliated Hospital of Wenzhou Medical University, Ruian, Zhejiang China; 5 Department of Oncology, Affiliated Hospital of Yangzhou University, Yangzhou, China

Retraction to: *Cell Death and Disease* 10.1038/s41419-021-03472-7, published online 15 February 2021

The Editors have retracted this article. After publication, concerns were raised regarding high similarity between A549 and SPCA1 si-NC images in Fig. 4F. The authors have been unable to share the underlying raw data upon request. Additionally, the SPCA1 cell line used in this study has been reported to be a derivative of HeLa cervical cancer cells [1], making it an unsuitable model for lung cancer.

The Editors therefore no longer have confidence in the presented data.

Mingming Jin does not agree with this retraction. None of the other authors have responded to any correspondence from the editor or publisher about this retraction.
